# Goal Orientations and Activation of Approach Versus Avoidance Motivation While Awaiting an Achievement Situation in the Laboratory

**DOI:** 10.3389/fpsyg.2018.01552

**Published:** 2018-08-28

**Authors:** Sigrid Wimmer, Helmut K. Lackner, Ilona Papousek, Manuela Paechter

**Affiliations:** ^1^Educational Psychology Unit, Department of Psychology, University of Graz, Graz, Austria; ^2^Section of Physiology, Otto Loewi Research Center, Medical University of Graz, Graz, Austria; ^3^Biological Psychology Unit, Department of Psychology, University of Graz, Graz, Austria

**Keywords:** achievement goal theory, task anticipation, approach/avoidance motivational system, EEG alpha asymmetry, energy mobilization

## Abstract

While some students try to give their best in an achievement situation, others show disengagement and just want to get the situation over and done with. The present study investigates the role of students’ tendencies for approach or avoidance motivation while anticipating tasks and the corresponding activation of the approach/avoidance motivational system as indicated by transient changes of EEG alpha asymmetry. Overall, 62 students (50 female; age: *M* = 23.8, *SD* = 3.5) completed a goal orientation questionnaire (learning goals, performance-approach, performance-avoidance, and work avoidance). They joined a laboratory experiment where EEG was recorded during resting condition as well as when students were anticipating tasks. Standard multiple regression analysis showed that higher values on performance-avoidance were related to a higher activation of the approach system whereas higher values on work avoidance were related to a higher activation of the avoidance system. Results question present assumptions about avoidance related goal orientations.

## Introduction

Nobody really likes exams. Individuals differ widely in how they cope with this kind of stressful situation. While some approach an exam with the intention of giving their best, others mainly want to avoid the situation. Achievement goal theory assumes that the choice for one of the above-mentioned behavior alternatives is influenced by stable personal dispositions: Goal orientations. The present study investigates to which degree goal orientations are related to approach or avoidance behaviors. It widens the commonly employed spectrum of research methodologies in the field by interlinking self-report measures via personality questionnaires with neurophysiological measurements of relevant changes in EEG alpha asymmetry in the brain’s prefrontal cortex.

### Approach and Avoidance Motivation and Goal Orientations

Motivation can be described as the energization and direction of behavior toward a stimulus (Elliot and Church, 1997; Wigfield et al., 2006). Behavior can be driven toward a desired stimulus (approach motivation) or away from an undesired stimulus (avoidance motivation).

Approach motivation comprises emotions, cognitions, and actions that are driven by the wish to achieve desirable results (e.g., good grades and feelings of competence) (Elliot and Covington, 2001; Lang and Bradley, 2008; Eder et al., 2013). It can be described as the energization of behavior which directs an individual toward a positive outcome. In contrast, avoidance motivation comprises emotions, cognitions, and actions that are driven by the wish to avoid an aversive situation or undesired consequences (e.g., punishment, threat, and failure) (Gray and McNaughton, 2000; Elliot and Covington, 2001) and can be described as the energization of behavior away from a negative stimulus.

Goals are the aim an individual strives for (Moskovitz and Grant, 2009). The expression goal “orientations” describes not only a network of feelings attributed to goals, but also the dispositional tendency to correspond to challenges in a certain behavior in specific situations (Elliot, 2007; Spinath, 2009). Therefore, theories on goal orientation address the distinction between approach and avoidance and assume that individuals are equipped with relatively stable dispositions, which imply a tendency either to achieve positive outcomes or avoid negative ones in the context of learning. Goal orientations refer to cognitive representations of the reasons for learning and achievement involvement (Elliot and Thrash, 2002; Elliot, 2007; Lackner et al., 2015) and explain the extensive variance in academic achievement as well as learning behaviors (Anderman et al., 2002; Spinath et al., 2002; Steinmayr et al., 2011; Harmon-Jones et al., 2013).

Research and theories on goals emerged to a large degree in the 1970s when several scientists, e.g., Ames, Dweck, Elliot, and Nicholls concentrated on achievement motivation in educational settings (Elliot, 2007; Payne et al., 2007). Within the various approaches of achievement goal theories, the trichotomous goal framework (Elliot and Harackiewicz, 1996) is one of the most commonly used models (e.g., Bipp et al., 2008; Xu et al., 2018). Even though researchers may employ different wordings they agree on a trichotomous goal framework with two approach and one avoidance related goal orientations: The approach goal orientations comprise two different motives for engagement in learning and achievement, i.e., learning goals (Dweck and Leggett, 1988; Elliot, 1999; also uses the term mastery goals) and performance-approach goals (Elliot and Church, 1997; also ego-involved goals, e.g., Nicholls, 1984; or ability goals, e.g., Ames, 1992). Learning goals describe the aim of mastering tasks and improving personal competencies (Elliot, 1999; Spinath et al., 2002). In contrast, performance-approach goals focus on the demonstration of own abilities in comparison to others and on competition in achievement situations (Elliot and Church, 1997). The third goal orientation in the trichotomous goal framework (Elliot and Harackiewicz, 1996) is performance-avoidance goal orientation, which focuses on avoiding failure and hiding an assumed lack of ability, and implies a tendency to avoid achievement situations.

However, based on observations of students in school or university settings, several theories on goal orientations have added work avoidance goal orientation as a fourth category (Nicholls, 1984; Dowson and McInerney, 2001; Spinath et al., 2002; Bipp et al., 2008). Work avoidance focuses on the goal of keeping the personal investment as low as possible (Nicholls, 1984; Elliot, 1999; Spinath et al., 2002). Students who score high on the work avoidance scale aim to avoid effort and to do just what is necessary for task accomplishment and not to increase own abilities or to compete with others (Dowson and McInerney, 2001; Bipp et al., 2008).

While some researchers (Elliot, 1999) criticize that this goal orientation differs conceptually from the other three types, others add it as a fourth type to the trichotomous model. Empirical evidence speaks for the inclusion of work avoidance which has a negative influence on learning and achievement and supplements the three other types, by the avoidance of effort (Dowson and McInerney, 2001; Pieper, 2003; King and McInerney, 2014).

Individuals usually display all four types of goal orientation. Within an individual, the four types differ from each other with regard to their importance and influence on behavior. When individuals encounter an achievement situation, goal orientations as relatively stable dispositions interact with the goal structures that a situation implies (Spinath and Stiensmeier-Pelster, 2003; Senko and Harackiewicz, 2005; Senko et al., 2011). Studies in the classroom yielded empirical evidence for all four goal orientations (e.g., Bipp et al., 2008; Senko et al., 2011). In the present study, learning goals, performance-approach goals, performance-avoidance goals, and work avoidance goals will be investigated to receive full information concerning students’ repertoire of goal orientations (Dowson and McInerney, 2001; Pieper, 2003).

### Goal Orientations and Coping With Stressful Academic Situations

Generally, approach goal orientations are associated with engagement, active coping tendencies, and the mobilization of energy in case of difficulties (Roth and Cohen, 1986; Dweck and Leggett, 1988; Fields and Prinz, 1997; Compas et al., 2001; Skinner and Zimmer-Gembeck, 2007). Moreover, both goal orientations are related to high competence expectancies, persistence, and a need for achievement (McClelland et al., 1953; Elliot and Church, 1997; Elliot et al., 1999).

In instructional settings, learning goals are favored over performance-approach goals. They are accompanied by intrinsic motivation and the use of active strategies of self-regulation. Learning goals are also linked with positive emotions such as enjoyment of learning, pride, task absorption, and well-being (Tuominen-Soini et al., 2008). They are not directly related to good grades because mastery learners strive for competence and not for external feedback; good grades are rather the result of an indirect positive relation via favorable learning behaviors (Elliot and Church, 1997).

Even though a performance-approach orientation is associated with active coping strategies and a need for achievement, it is also accompanied by less desirable motivational tendencies and learning behaviors. Performance goals are more strongly related to extrinsic than to intrinsic motivation. In comparison to a learning goal orientation, a performance-approach orientation is more vulnerable in case of a setback or negative feedback, especially when students have a lower self-concept or doubt their accomplishment of a given task (Meece et al., 1988; Grant and Dweck, 2003). Therefore, performance approach orientation has also been linked to test-anxiety, negative affect, and stress (Tuominen-Soini et al., 2008). Midgley et al. (2001) point out that the long-term effects of a performance-approach are most beneficial in highly competitive environments and/or for students with high self-concept in a domain. If in contrast individuals with performance goals assume that they lack the ability to reach their goal, they tend to fall into behavioral patterns of helplessness, and develop maladaptive cognitions and behaviors that result in poor achievement outcomes (Dweck and Leggett, 1988; Spinath and Stiensmeier-Pelster, 2003).

The maintenance and success of both approach orientations generally depends on access to or the possession of social (e.g., social/family support), material, and personal (e.g., abilities, persistence, and concentration) resources (Diener and Fujita, 1995; Schnelle et al., 2010) as well as on a positive (yet realistic) self-concept of the own abilities (Spinath and Stiensmeier-Pelster, 2003).

Performance-avoidance goal orientation is regarded as undesirable in academic contexts. It is associated with low competence expectations, low academic aspirations, fear of failure, and low intrinsic motivation (Elliot and McGregor, 2001; Spinath et al., 2002). Students who score high on performance-avoidance are also prone to test anxiety and tenseness and show a tendency toward helplessness patterns in academic challenges (Elliot and Church, 1997; Elliot et al., 1999; Grant and Dweck, 2003; Spinath and Stiensmeier-Pelster, 2003). Overall, research associates performance-avoidance goals with lower capabilities and lower success in coping with difficult life situations in general (Roth and Cohen, 1986; Fields and Prinz, 1997; Compas et al., 2001; Skinner and Zimmer-Gembeck, 2007). Elliot et al. (2011) use the term “avoidance coping” (p. 624) to describe how individuals’ avoidance goals lead to the selection and use of avoidance strategies to deal with difficult situations (e.g., in academic contexts). They conclude that the use of avoidance strategies often has a negative impact on individuals’ well-being.

Work avoidance is regarded as the least desirable of the four goal orientations. For students with a high work avoidance, “success” is defined in terms of minimal work expenditure and not according to any external or individual measure of competence. It is also negatively correlated with satisfaction with learning and deep level processing strategies; students pursuing this goal orientation assess their skills rather negatively (Nicholls et al., 1985; Nolen, 1988). Work avoidance goals are also associated with less involvement, lower grades, anxiety, lower self-esteem, and low well-being (Tuominen-Soini et al., 2008; King and McInerney, 2014).

### Methodological Issues in Investigating Goal Orientations

The influence of goal orientations on emotional or motivational states, effort, or learning strategies has to date mostly been investigated by self-report methods (e.g., Elliot et al., 1999; Grant and Dweck, 2003; Schnelle et al., 2010; Schwinger et al., 2012; King and McInerney, 2014). However, while individuals often clearly know how they usually behave or how they feel in a specific situation (Paulus and Vazire, 2007), the accuracy of self-reports may suffer from inaccuracies and response bias.

The present study uses validated self-report measurements but also takes into consideration the neural underpinnings of approach/avoidance motivation by using EEG alpha asymmetry measurements in the brain’s prefrontal cortex. Even though the application of neuroscientific methods for the investigation of motivational processes in more applied contexts is just at its beginning (Harmon-Jones and van Honk, 2012; Di Domenico and Ryan, 2017), various reasons speak for them. First and rather obviously, attitudes, experiences, and behavior are mediated by the brain and a complete understanding of motivational processes profits from a cross-disciplinary linkage of different sources of information and data. Furthermore, the inclusion of neuroscientific methods allows the investigation of internal processes that are not accessible by self-reports or behavior observations (Di Domenico and Ryan, 2017). Measures of brain activity allow to capture actual motivational states as well as rapid changes in motivational responses that cannot be accessed by self-reports or that are not even available in consciousness (Harmon-Jones and van Honk, 2012).

Against this background, the present study pursues a cross-disciplinary approach and combines measurements of EEG frontal asymmetry with valid self-reports. Self-reports where used to measure goal orientations as dispositions whereas the EEG was used to capture the participants’ actual motivational state in a relevant context.

EEG frontal asymmetry is related to increases in approach-related and avoidance-related behavior (Davidson, 1998; Harmon-Jones et al., 2010; see also Fox and Reeb, 2008). Researchers here refer to an approach/avoidance motivational system, which is one of the most acknowledged concepts in psychology (Elliot and Covington, 2001). Given relevant situational contexts, transient changes in EEG alpha asymmetry in prefrontal cortex represent an objective correlate of the relative activation of the approach and avoidance motivational systems at a given moment (Harmon-Jones et al., 2010; Papousek et al., 2014, 2018).

This was confirmed by a number of empirical studies in which avoidance or approach oriented motivational states were evoked (Sobotka et al., 1992; Shankman et al., 2007, 2011; Flo et al., 2011; Price and Harmon-Jones, 2011). Conditions that encourage the approach motivational system produced a transient lateral shift toward relatively greater activity in the left versus right prefrontal cortex (indexed by attenuated EEG alpha band activity), whereas conditions that encourage the avoidance system produced a shift to the right. Moreover, depending on the relative activation of the two motivational systems, individuals greatly differ in their prefrontal EEG alpha asymmetry responses when confronted with the same conditions (see, e.g., Papousek et al., 2014, 2018). These inter-individual variations are expected to index the proneness for approach tendency versus avoidance tendency dominated responses during specific emotionally salient events (Coan et al., 2006).

The validity of this interpretation was confirmed by a number of studies showing relationships between inter-individual differences in the EEG alpha asymmetry response to specific situations or stimuli and traits which indicate a predisposition for high or low approach- or avoidance-oriented motivation in these specific situations. Examples are social anxiety, greater like-ratings of objects, detached and antagonistic personality traits, melancholia, neuroticism, borderline personality disorder, and specific genetic predispositions (Davidson et al., 2000; Gable and Harmon-Jones, 2008; Harmon-Jones and Gable, 2009; Cole et al., 2012; Papousek et al., 2013, 2018; Wacker et al., 2013; Beeney et al., 2014; Uusberg et al., 2015; Liu et al., 2016).

Taken together, analysis of inter-individual differences as they relate to the direction and magnitude of the prefrontal EEG alpha asymmetry response to specific stimulation provides the opportunity to gain an objective, well-validated indicator of an individual’s proneness for the relative activation of approach versus avoidance motivation in the specific context it was recorded (Papousek et al., 2018). The extent to which a transfer of conclusions to related real-life situations is justified depends on the relevance of the context established in the laboratory to these real-life situations. In the present study, a realistic achievement situation was set up for this purpose.

### Research Questions and Methodology of the Present Study

The present study investigated firstly and foremost to which degree goal orientations as dispositions are related to the actual relative activation of the approach, respectively, avoidance motivational system in an achievement context, that is, while participants were anticipating statistics tasks. Four different types of academic goal orientations are used in this study to receive full information concerning students’ motivation beyond learning and performance goal orientations (Pieper, 2003; King and McInerney, 2014). According to previous findings, statistics tests have stressful and negative connotations for students (e.g., Paechter et al., 2017). Anticipation of a stressful task elicits affective and physiological responses comparable to those observed during exposure to the task itself. The use of anticipation conditions is a conventional method in psychophysiological investigations for studying physiological effects of emotional/motivational demands without contaminating the measures by the effects of the behavioral demands of the task such as speaking, motor responses, or task-related cognition (e.g., Feldman et al., 2004).

Whereas previous research on achievement goal theory has focused on the assessment of goal orientations and their antecedents or their effects (Elliot and Murayama, 2008), the present study examines the impact of dispositional goal orientations on the actual relative activation of approach versus avoidance motivation directly while awaiting a stressful task. While goal orientations have often been used as predictors for outcome variables like interest or performance (Elliot and Church, 1997; Zusho et al., 2005), in the present study, they are used to predict relative changes in the participants’ motivational state. Regression analyses were applied to illustrate the impact of the stable goal orientation on actual changes in the motivational state due to anticipation of aversive achievement tasks.

Secondly, it was investigated to which degree the goal orientations are related to differences in negative emotions such as tenseness. As it was discussed before, different goal orientations should especially be related to differences in negative feelings in the respective achievement situation (e.g., Elliot et al., 1999; Tuominen-Soini et al., 2008; King and McInerney, 2014). Lastly, in order to take task-related characteristics and individual variables into account, gender, difficulty ratings of the tasks, and effort were correlated with goal orientations and the EEG alpha asymmetry response.

## Materials and Methods

### Participants

Sixty-two psychology students completed the experiment with all required data (12 men and 50 women). Participants were between 20 and 38 years old (*M* = 23.8, *SD* = 3.5) and all of them right-handed (assessment by a standardized hand skill test). Individuals who reported having a neurological or psychiatric disorder or the use of psychoactive medication did not take part. Participants were to refrain from alcohol 12 h prior to the study, and from coffee and other stimulating beverages 2 h prior to their lab appointment, and to come to the session well rested. The study was performed in accordance with the American Psychological Association’s Ethics Code and the 1964 Declaration of Helsinki. It was also approved by the local authorized ethics committee of the University of Graz. Information about the study and conditions for participation were spread in different lectures at the start of term. Participation in the study was voluntary and written informed consent was obtained from all participants. All participants gave their written informed consent to participate and to confirm that their data were used in an empirical study.

### Academic Goal Orientations

The student version of the scales for the Assessment of Learning and Performance Goals (SELLMO-ST; Spinath et al., 2002) were used to assess academic goal orientations as a trait. It comprises 31 statements starting with “In my studies it is important for me...,” rated on 5-point Likert scales ranging from “totally disagree” (1) to “totally agree” (5). Responses are grouped into four scales, each representing a goal orientation: Learning (8 items, e.g., “... to learn something interesting,” Cronbach’s alpha in the current study α = 0.76); performance-approach (7 items, e.g., “... to get better grades than others,”, α = 74); performance-avoidance (8 items, e.g., “... to hide if I know less than others,” α = 0.89); work avoidance (8 items, e.g., “... to finish my studies with little effort,” α = 0.86).

### Measurement of Tenseness

Participants assessed their tenseness before the start of the experiment as well as after completing the tasks by a standardized, verbally anchored 17-point bipolar rating scale for mood and feelings (KUSTA; Binz and Wendt, 1986). The poles of the tenseness-scale are labeled “restless, nervous, and tense” to “calm, relaxed, and balanced.” The scale differentiated well between individuals and was sensitive to small short-term changes in emotional states in previous investigations (e.g., Papousek et al., 2010, 2011).

### Subjective Ratings

In order to assess the influence of other individual and task-related characteristics, upon completion, participants rated their perception of the overall difficulty of the tasks as well as regarding how hard they had tried to solve them. Participants rated perceived difficulty (“How difficult has it been for you to solve the tasks?”) and effort (“How hard have you worked to solve the tasks?”) on 17-point Likert scales ranging from 1 (not at all) to 17 (extremely). See **Table 1** for detailed results.

**Table 1:** Mean, standard deviations, minimum, maximum, and *n* for the investigated variables.

Variable	*n*	*M*	*SD*	*Min.*	*Max.*
**SELLMO-ST scales**					
Learning (1 low to 5 high)	62	4.5	0.4	3.3	5.0
Performance-approach (1 low to 5 high)	62	3.0	0.6	1.3	4.6
Performance-avoidance (1 low to 5 high)	62	2.1	0.8	1.0	3.6
Work avoidance (1 low to 5 high)	62	1.6	0.6	1.0	3.1
**Subjective ratings**					
Effort (1 not at all to 17 extremely)	62	10.7	4.1	1	17.0
Perceived task difficulty (1 not at all to 17 extremely)	62	8.9	4.5	1	17.0
Tenseness before task (17 tense to 1 relaxed)	62	6.9	3.9	2.0	14.0
Tenseness after task (17 tense to 1 relaxed)	62	9.0	4.1	1.0	16.0
**EEG alpha asymmetry response**					
LC anticipation – LC rest	62	-0.48	5.78	-12.01	17.64

### EEG Recording and Quantification

The EEG was recorded from 19 channels according to the international 10–20 system, using a Brainvision BrainAmp Research Amplifier (Brain Products; sampling rate 500 Hz, resolution 0.1 μV) and a stretchable electrode cap, referenced to the nose and re-referenced offline to a mathematically averaged ears reference (Hagemann, 2004). Impedance was kept below 5 kΩ for all electrodes. The most common site in the brain for lateral shifts of EEG alpha asymmetry in the context of approach versus avoidance motivation is the dorsolateral prefrontal cortex in the region of the EEG electrode positions F3 and F4. These electrodes were used for obtaining the EEG alpha asymmetry response to the performance challenge (see Papousek et al., 2013, 2014, 2018). All data were visually inspected to eliminate intervals in which ocular or muscle artifacts occurred. Power spectra (epoch length 1 s, overlapping 50%, Hanning window) were averaged across all artifact-free intervals for all individuals.

Following the common approach in the field, power within the alpha frequency band (8–12 Hz) was used for the analyses. Laterality coefficient (LC) indexing relative right- versus left-sided activation were computed as follows: LC = [(R - L)/(R + L)] × 100. This asymmetry ratio is equivalent to another common EEG asymmetry metric (lnR - lnL), with which it is virtually perfectly correlated considering the small physiologically expectable range of relative differences between the EEG alpha power at two homologous electrodes (see Allen et al., 2004; Papousek et al., 2018).

Consistent with existing research (e.g., Papousek et al., 2014, 2018), and to obtain an index of the EEG alpha asymmetry response, linear regressions were conducted using LC during the reference condition (resting condition) to predict LC during anticipation of the statistics exam questions to calculate residualized change scores. Thereby it was ensured that the analyzed residual variability was due to the stimulation, and not to individual differences in baseline levels, and the measurement error inherent in the use of repeated measures of the same kind was controlled (e.g., Steketee and Chambless, 1992; Linden et al., 1997). Higher negative values are assumed to indicate a greater relative increase of right- versus left-sided activity (inverse of alpha) in the dorsolateral frontal region. According to the literature reviewed above, higher positive values are anticipated as indicating greater relative activation of approach versus avoidance motivation, while higher negative values are assumed to indicate greater relative activation of avoidance versus approach motivation.

### Procedure

Participants completed the hand skill test, the SELLMO-ST, the KUSTA and several other questionnaires that did not directly pertain to the present research question. Psychophysiological recordings were conducted in an EEG-laboratory, where participants were seated in an acoustically and electrically shielded laboratory room. All instructions were given via a LCD-screen in the laboratory room. Additional information was provided by the investigator-in-charge before the recordings started and before the statistics tasks were presented. The investigator-in-charge was outside the examination room. During the EEG recordings participants were monitored by a camera.

EEG-recordings started with a 2 min baseline recording, i.e., participants were instructed to relax, sit quietly, and to fix their eyes on a solid green circle on the screen.

After baseline recordings participants were informed that they would next receive statistics tasks (which are learning contents for the Bachelor degree). They were asked to solve these tasks as best as possible and to answer orally. To enhance the personal relevance of the tasks, a text on the screen informed the participants that the tasks resemble those used in the admission test for the Master’s degree. Participants were then again asked to relax, sit quietly, and to fix their eyes on the green circle for 2 min (EEG-recordings for anticipation period). After that the investigator-in-charge informed orally that the statistics tasks now would be presented and asked to answer them verbally. Then the three statistics tasks were posed; for each task, participants had 30 s to read and 70 s to answer (e.g., explain the error type 1 and type 2. Give an example when error type 2 is more important than error type 1). Overall this took 5 min time.

After completion, participants indicated the perceived difficulty of the tasks as well as how hard they had tried to find the answers. Finally, they filled in the KUSTA tenseness scale again.

## Results

Descriptive statistics of goal orientations, estimations of effort, tenseness, and LC are shown in **Table 1**.

The first research question was tested with a standard multiple regression analysis using the four SELLMO-ST scales as predictors and the EEG alpha asymmetry response to the imminent performance situation as the dependent variable [*F*(4,57) = 2.9, *p* = 0.032, *R*^2^ = 0.17]. The effect size of Cohen’s *f*^2^ = 0.205 speaks for a medium effect (Selya et al., 2012). Details are listed in **Table 2**. While awaiting the performance situation, participants with higher levels of work avoidance showed more relative activation of avoidance versus approach motivation. Participants scoring higher on performance-avoidance goals showed more relative activation of approach versus avoidance motivation. Performance-approach goals and learning goals did not show significant relationships to the EEG alpha asymmetry response. **Table 3** shows the intercorrelations (Pearson correlations) among the SELLMO-ST subscales.

**Table 2:** Goal orientations regressed on laterality coefficient (LC).

SELLMO-ST scales	ß	*sr*	*p*
Learning	0.07	0.06	0.610
Performance-approach	–0.04	–0.04	0.765
Performance-avoidance	0.45	0.37	0.003
Work avoidance	–0.34	–0.26	0.035

*Higher negative values of the EEG alpha asymmetry response denote a greater relative increase of right- versus left-sided brain activity (inverse of alpha), i.e., relative activation of avoidance versus approach motivation. Higher positive values denote relative activation of approach versus avoidance motivation. β = Beta weights in the regression analysis; sr, semi-partial correlations controlling for the other three subscales of the SELLMO-ST.*

**Table 3:** Bivariate correlations between SELLMO-ST scales of goal orientation.

SELLMO-ST scales	2	3	4
1. Learning	–0.04 (0.744)	–0.15 (0.262)	–0.40 (0.001)
2. Performance-approach		0.32 (0.012)	0.24 (0.064)
3. Performance-avoidance			0.53 (0.000)
4. Work avoidance			–

*Note: *r* (*p*).*

The second research question was tested with two standard multiple regression analyses using the four SELLMO-ST scales as predictors and the assessments of tenseness as dependent variables [tenseness before (*F*(4,57) = 4.4, *p* = 0.004, *R*^2^ = 0.24); after (*F*(4,57) = 3.4, *p* = 0.015, *R*^2^ = 0.19)]. The effect size of Cohen’s *f*^2^ = 0.235 speaks for a medium effect (Selya et al., 2012). Details are listed in **Table 4**. While tenseness was significantly lower in participants with higher levels of learning and work avoidance goals before the start of the experiment as well as afterward, the opposite was observable for participants high on performance-avoidance goals. Participants scoring high on performance-avoidance goal orientation reported higher levels of tenseness before and after completing the tasks. Bivariate correlation between the two affect measurements was *r* = 0.50, *p* < 0.000.

**Table 4:** Goal orientations regressed on tenseness before and after task completion.

	Before task completion			After task completion		
SELLMO-ST scales	*ß*	*sr*	*p*		*ß*	*sr*	*p*
Learning	–0.44	–0.40	0.00		–0.25	–0.23	0.06
Performance-approach	–0.07	–0.06	0.59		–0.04	–0.04	0.74
Performance-avoidance	0.35	0.29	0.02		0.46	0.38	0.00
Work avoidance	–0.42	–0.33	0.01		–0.25	–0.20	0.10

In order to assess and to control for effects of task-related, individual variables further correlations were calculated. Participants’ ratings of how hard they tried to answer the questions was not related to the EEG alpha asymmetry response (*r* = 0.19, *p* = 0.143). It showed a positive correlation with the difficulty rating (*r* = 0.33, *p* = 0.009). No significant correlations were observed between this rating and any of the goal orientations (*r* = -0.18, *p* = 0.152; *r* = 0.02, *p* = 0.888; *r* = 0.08, *p* = 0.551; *r* = 0.17, *p* = 0.180). There were no gender differences in any of the goal orientations, nor in the EEG alpha asymmetry response (in independent *t*-tests, all *p*’s > 0.35).

## Discussion

The present study investigated to which degree the four types of goal orientations as dispositions are related to the actual relative activation of the approach versus avoidance motivational system in a performance challenge situation. In the laboratory setting; an important task phase was captured: activation due to the anticipation of tasks.

Contrary to what would be expected from avoidance motivation, e.g., low aspirations, difficulties in mobilizing energy, or helpless behavior (Elliot and Covington, 2001; Elliot and McGregor, 2001), higher values of performance-avoidance goal orientation were related to a higher activation of the approach motivational system. At first glance, this result contradicts studies in which performance-avoidance was related to disengaged behavior and withdrawal from aversive situations such as academic challenges (e.g., Obrist et al., 1978; Carver et al., 1989; Elliot and Church, 1997; Steele-Johnson et al., 2000; Spinath et al., 2002).

An analysis of the demands of the situation in the laboratory and its goal structures might help explain this result. Participants were faced with aversive statistics tasks and a situation that, from a performance-avoidance perspective, would advise withdrawal. In the experimental setting, however, withdrawal by calling off the ongoing experiment or by avoiding effort and failing the tasks would have yielded negative consequences such as embarrassment in front of the investigator-in-charge and/or depreciating evaluations of one’s task performance. The goal of avoiding failure or embarrassment could only be achieved by engagement coping, i.e., by actively dealing with the stressor, and not by disengagement and escaping the task (Carver and Connor-Smith, 2010). Altogether, performance-avoidance orientation used the prevention of a negative outcome as a hub of approach activity and taking on the stimuli (see also Harmon-Jones et al., 2013). As such, performance-avoidance orientation was still related to negative emotions. Performance-avoidance goal orientation as a disposition contributed to higher levels of tenseness both prior to and after task performance.

The combination of approach and avoidance within a situation, i.e., approaching in order to avoid, is an important mechanism for coping because it enables individuals with aversive dispositional tendencies to adjust behavioral tendencies toward the activation of resources. Whether a performance-avoidance tendency leads to an approach motivation also depends on the expectancy of success in case of the intended behavior alternative (Macher et al., 2013; Paechter et al., 2017). Altogether, the results suggest that performance-avoidance goals do not solely focus on avoiding challenges. They furthermore emphasize the beneficial potential which the activation of this type of goal orientation may bear.

Of the four goal orientations, only proneness to work avoidance was related to an activation of avoidance over approach motivation. This result corresponds to a laboratory study by Lackner et al. (2015) in which work avoidance was related to lower levels of cardiovascular activation during task processing indicating less approach-oriented/active coping with the challenge. The authors, however, found no relationship between cardiac activation during task processing and the three other goal orientations.

In the present study, work avoidance was also related to lower levels of tenseness prior to the tasks and with a statistical tendency toward tenseness after the tasks. Disengagement and indifference toward the demands of the situation characterize this type of goal orientation. According to motivational intensity theory (Brehm and Self, 1989) individuals mobilize only as much resources as are needed for goal attainment. In addition, importance of success and task difficulty are crucial factors for the degree of mobilization. The activation of avoidance over approach motivation and the lower degrees of tenseness suggest that students scoring higher on work avoidance did not attach much importance to task achievement. With this in mind, the results speak in favor of the concept of work avoidance as a reflection of reduced engagement and indifference against goal achievement. However, in a laboratory situation, an early disengagement from the task has hardly any consequences, while on the other hand, in many real-life achievement situations this kind of behavior could be dysfunctional or unacceptable.

Against expectations, both approach goal orientations were not related to relative activation of the approach motivational system. The dispositional pursuit of learning goals or performance approach goals may have been of lower importance in the study. Success or failure did not have any further consequences concerning individual advancement or self-affirmation because the laboratory achievement situation did not provide any opportunity to increase the own knowledge, compete, or demonstrate own abilities. Hence, the habitual performance goal orientations in academic daily life may not have been unfolded themselves in the laboratory task as may be suggested by studies in which performance goals were experimentally manipulated immediately before task performance (Chalabaev et al., 2009). An experimental paradigm that activates learning and performance-approach goals probably needs to focus more on the individual benefits of the performance.

A final and important result concerns the subjective ratings of task difficulty and effort. There was a small yet significant positive relationship between difficulty ratings with the motivational response. Participants who experienced the tasks as being more difficult showed a relative activation of approach versus avoidance motivation, which corresponds with motivational intensity theory (Brehm and Self, 1989). In contrast, the subjective ratings of effort did not correlate significantly with motivational responses. There are different possible explanations for this result. On the one hand, it might be difficult for individuals to precisely assess inner states such as effort without bias. On the other hand, students might use different internal frames of references and compare the effort they invested for the laboratory tasks with similar situations in the past. So different individual frames of references might attenuate and/or vary the differences between participants.

### Implications and Limitations of the Study

The results have implications for future research strategies, as they emphasize the benefits of a combination of data gained by self-reports and validated physiological measures. Current motivational tendencies are difficult to assess using self-report, especially while individuals prepare themselves mentally for task completion or while they complete a task. Physiological indicators have the advantage that they are not consciously controlled, bypass the regulatory controls exerted on overt behavior, and are independent of participants’ ability or willingness to accurately report their own experience. In addition, more subtle or fleeting changes can be examined that are difficult to capture with retrospective self-report (Blascovich et al., 2004; Schwerdtfeger, 2004; Vick et al., 2008; Allen et al., 2012). One may ask to which degree findings from the laboratory situation can be transferred to learning and achievement situations in real life. This question can be answered by looking at the characteristics and the consequences of behavior alternatives in the laboratory situation and by comparing them with characteristics of a real-life situation. For example, one might assume that individuals with high scores on work avoidance would similarly behave in a real-life situation with few incentives and behavioral consequences.

Implications of the present study also concern the framework of goal orientations. Approach motivation is not related to appetitive stimuli only. Unpleasant experiences like goal frustration may evoke the impulse to go even toward a negative stimuli, e.g., to recapture a goal (Carver and Harmon-Jones, 2009; Harmon-Jones et al., 2013). The results from this study suggest that performance-avoidance goal orientation is related to approach behavior and, thus, may initiate an approach “in order to avoid” (Elliot, 2006, p. 114). This result not only contradicts negative assumptions about performance-avoidance goal orientation. In emphasizing its potential of mobilizing energy, it furthermore questions the term of “performance-avoidance.” As a result, performance-avoidance goals should instead be analyzed with a view of the situation, its goal structures, and consequences of behavior alternatives.

Moreover, the present findings suggest that not only why students are motivated (e.g., to successfully accomplish something and/or avoid failure), but also to which degree students are motivated to invest effort should be included in a framework of goal orientations. Results imply that students scoring higher on work avoidance reduce their resources and withdraw during the anticipation of tasks itself. Work avoidance appears to induce passive coping, which prevents students from engaging in the tasks. The trichotomous goal orientation model focusses on motivated students and investigates the effects of their reasons for being motivated. In these models, work avoidance is not considered as a type of motivation and is not included (e.g., Elliot, 1999). However, in the present study, work avoidance appears to represent the only “pure” avoidance motivation factor. Since motivation can also result in the intentional omission of an action (Gredler, 2001), we assume that a reduction in effort as indicated by a lack of cardiac activation (Lackner et al., 2015), or a shift toward greater relative right frontal activation as observed in the present study (each of which are related to work avoidance) indicate that work avoidance represents an important factor in students’ goal orientation (Pieper, 2003).

The results also have implications for learning, instruction, and diagnosis of learning difficulties. Counseling or coaching, in case of learning difficulties, should take dysfunctional goal orientations, such as work avoidance into account. Also, teachers and instructors should be aware of goal orientations such as dispositions and take them into consideration when designing instruction, developing tests, or concerning the support they provide (King and McInerney, 2014). The results recommend reducing tenseness in those students scoring high on performance-avoidance in test situations (e.g., by positive feedback that relates to skills and abilities) and promoting approach-to-avoid or, in the case of high levels of work avoidance, finding incentives that emphasize the personal value of learning. Also, parents should be aware that they influence and shape their children’s welcome or not-so-welcome goal orientations (Xu et al., 2018).

Although the present results may not be fully transferable to real achademic achievement situations, they provide information on how students’ approache challenges. Students’ (beneficial or dysfunctional) tendency to engage or to withdraw according to their dispositional goal orientations – even if the task was merely announced – could clearly be observed. As mentioned, further research should consider a setting with higher ecological validity that offers the possibilty to trigger approach- as well as avoidance-related goal orientations. Moreover, prospective investigations on academic goal orientations may benefit from including more covariates such as self-efficacy in the study of relationships between goal orientations and emotional/motivational responses in achievement situations.

## Author Contributions

All authors listed have made a substantial, direct and intellectual contribution to the work, and approved it for publication.

## Conflict of Interest Statement

The authors declare that the research was conducted in the absence of any commercial or financial relationships that could be construed as a potential conflict of interest.
